# Towards an Epistemology of ‘Speciesist Ignorance’

**DOI:** 10.1007/s11158-024-09656-0

**Published:** 2024-02-26

**Authors:** Emnée van den Brandeler

**Affiliations:** Department of Philosophy, University of Basel, Steinengraben 5, 4051 Basel, Switzerland

**Keywords:** Moral ignorance, Speciesism, Animal ethics, Group ignorance, Human privilege

## Abstract

The literature on the epistemology of ignorance already discusses how certain forms of discrimination, such as racism and sexism, are perpetuated by the ignorance of individuals and groups. However, little attention has been given to how speciesism—a form of discrimination on the basis of species membership—is sustained through ignorance*.* Of the few animal ethicists who explicitly discuss ignorance, none have related this concept to speciesism as a form of discrimination. However, it is crucial to explore this connection, I argue, as ignorance is both an integral part of the injustice done to animals as well as an obstacle to improving their treatment. In order to adequately criticize sustained structural speciesism and injustices towards animals, I develop an epistemological account of ‘speciesist ignorance’. I begin by defining and distinguishing between individual and group-based accounts of speciesist ignorance. I argue that humans, taken as a group, enjoy a position of privilege, which allows them to comfortably remain ignorant of their participation in collective wrongdoings towards animals. Additionally, I point out that speciesist ignorance is structurally encouraged and thereby maintains the dominant view that the human-animal-relationship, as it stands, is just. In sum, this article lays the groundwork for a social epistemology of speciesist ignorance. In particular, it informs further debate about individual and institutional epistemic duties to inquire into speciesism and to inform the public, about the moral culpability of ignorant actions, and about effective animal advocacy and policy which actively rejects speciesist ignorance.

## Introduction

Many animal ethicists have argued that speciesism is a form of discrimination—one that is made on the basis of species membership. A common thread running through animal ethics and the animal rights movement is that once people recognize such speciesism, a trend towards collective political action and anti-speciesist governance will logically ensue.^1^ Unfortunately, there seems to be widespread *ignorance* about this form of discrimination—an ignorance, that amounts to more than a simple non-knowing. Peter Singer suggests that ‘[i]gnorance […] is the speciesist’s first line of defense. Yet it is easily breached by anyone with the time and determination to find out the truth' (2015, p. 314). It seems plausible that ignorance is strategically used to avoid moral blame and its repercussions, since the blissfully ignorant speciesist benefits immensely from using animals, i.e., for research, food, entertainment, and so on. And yet, the topic of ignorance *itself* has not received much attention from animal ethicists. Neither has *speciesism* received attention from epistemologists who discuss the theoretical and political role of ignorance. To put this into perspective, the role of ignorance in discriminatory practices *has* been discussed for other forms of discrimination, such as sexism (e.g., see O’Neill 2021; Pateman 2016), and racism (e.g., see Bain 2018; Martín, 2021; Mills 1997, 2007; Sullivan 2007; Sullivan and Tuana 2007).

In this article, I explore the connections between ignorance and speciesism from a philosophical perspective. First, I explain why it is desirable to include an epistemology of ignorance into the speciesism debate. Second, I discuss individual epistemic conceptions of ignorance, and provide a definition for individual speciesist ignorance. Third, I argue that speciesist ignorance also functions as a group-based form of ignorance, and provide a definition for group-based speciesist ignorance. Fourth, I identify which social processes and institutions encourage ignorance, highlighting some of the social-epistemic dimensions of speciesist ignorance. Fifth, I propose that this conception of speciesist ignorance explains how injustices towards animals are perpetuated through social institutions and interactions. Lastly, I conclude that amid omnipresent speciesist behaviour and speciesist governance, the implications of speciesist ignorance must be further considered within the fields of ethics and epistemology to ensure moral progress in the human-animal-relationship.

## Speciesism and Ignorance

The concept of speciesism has played an important role in the field of animal ethics for delineating the moral status of animals. As first conceptualized by Ryder (1975)—and later popularized by Peter Singer (Singer 2009, 2015)—speciesism is analogous to other –isms, such as sexism and racism (Singer 2009). For the sake of my argument, I assume that speciesism is indeed a form of discrimination, and can therefore constitute a moral wrong. When discrimination among individuals happens on the basis of a morally superfluous trait—such as skin colour, sex, or cognitive intelligence—it is morally objectionable. Therefore, speciesism is best defined as ‘discrimination based on species membership’ (Jaquet 2022, p. 934). Specifically, for the purposes of this article, I rely on the definition of speciesism^2^ as ‘discrimination against those who are not classified as belonging to one or more particular species’ (Horta 2010, p. 247).

In the remainder of this article, I will sidestep the debate about speciesism and assume that the average speciesist is morally ignorant—i.e., ignorant about her anti-speciesist moral obligations towards animals. Speciesism is so normalized, that one can indeed quickly disregard how deeply it is embedded in our behaviour as well as in the policies governing the treatment of animals. However, the connection between speciesism and *ignorance* has received little attention in the literature.^3^ The very few animal scholars who have discussed ignorance primarily frame it as an *individual strategy* to avoid moral blame—and thus only consider a very narrow understanding of ignorance. Namely, they argue that wilful ignorance enables individuals to continue benefitting from animal use, while simultaneously protecting against their moral conscience (Onwezen and van der Weele 2016; Williams 2008). Peter Singer has also alluded to this motivation: ‘Ignorance has prevailed so long only because people do not want to find out the truth. "Don’t tell me, you’ll spoil my dinner" is the usual reply to an attempt to tell someone just how that dinner was produced’ (Singer 2015, p. 314). In Singer’s example, the speciesist wishes to avoid moral blame and its repercussions for her behaviour. Indeed, meat-eaters tend to defend their behaviour by claiming that meat production is normal, that it is natural, that it is necessary for our health, or that it is not seriously harmful to animals or morally wrong (Abbate 2021; Hopwood and Bleidorn 2019; Joy 2020). By staying ignorant, the speciesist can unquestioningly and blindly continue her wrongdoings towards animals.

On the conceptual level, the connection between speciesism and ignorance is much more complicated than a simple non-knowing about speciesism and one’s own speciesist ways, precisely because of the vested interests with respect to using animals. As such, the field of animal ethics requires a deeper understanding of the *epistemic dimensions* of speciesism.^4^ To this end, I will introduce an *epistemology of ignorance* into the animal ethics debate. Indeed, it is necessary to conceptualize speciesist ignorance as a special form of ignorance, I suggest, because the human-animal-relationship is *epistemically specific*: this epistemic relationship is especially fragile in comparison with inter-human relationships. To be sure, epistemic uncertainties also play into inter-human relationships, but they are arguably even more prevalent across species. For instance, there exist many uncertainties about the target of ignorance with respect to animals (i.e., what animals’ interests and desires consist in); crucially, these epistemic uncertainties are misused by humans to undermine animals’ abilities and subjective experiences. And it is this misuse of epistemic uncertainties in the human-animal-relationship, I claim, that makes our ignorance of a specific type, requiring its own conceptualization in the philosophical debate.

On the theoretical level, a conception of speciesist ignorance can perform various functions that make it instrumental to the animal ethics debate. First, it improves our understanding of how (speciesist) ignorance influences injustices towards animals. This conception describes the speciesist status quo with greater accuracy, for instance, by explaining why speciesist ignorance (and the consequential wrongdoings) are so widespread. Second, a conception of speciesist ignorance urges us to correctly allocate blame, by considering when ignorance about moral wrongdoings may provide an excuse. If I do not know that my action was wrong, it is questionable whether I am blameworthy for this wrongdoing. Under what circumstances and to what degree may speciesist ignorance excuse blame for common actions, such as eating meat? Third, a conception of speciesist ignorance prompts us to better appreciate how the phenomenon of ignorance comes about, and thereby, to devise effective advocacy strategies and policies to discourage this form of ignorance. Fourth, a clear conception of speciesist ignorance may change our view of what injustices towards animals consist in. For instance, the injustices which animals suffer at the hands of humans may have an important, hitherto overlooked epistemic dimension. Further, if speciesist ignorance turns out to *perpetuate* the injustices towards animals, it may also, more fundamentally, constitute an intrinsic *facet* of injustices towards animals.

On the practical level, recognizing speciesist ignorance as such is especially important, given the high moral stakes of the corresponding speciesist practices. Arguably, our speciesist ways could continue indefinitely for as long as our mistreatment of animals is not widely viewed as a moral issue at all. Such widespread moral ignorance significantly impedes any efforts to implement or even consider the political reforms that would be required to achieve just inter-species societies (Milligan 2015).

For all of these reasons, the animal ethics debate stands to be enriched by an epistemology of speciesist ignorance. In the following section, I illustrate how ignorance has been discussed in the animal ethics literature so far and suggest how this concept’s relation *to speciesism* could be further explored. Since ignorance is straightforwardly understood as an epistemic stance of *individuals*, let us start there.

## What is Ignorance?

The concept of ignorance admits of several distinctions.^5^ To begin, one can distinguish between *propositional ignorance* (that p is true or untrue), *objectual ignorance* (about objects, persons, or knowledge by acquaintance), and *practical ignorance* (how to do something) (Nottelmann 2016). For instance, some people may be ignorant of the fact that a certain animal species is sentient, of the existence of *foie gras* farms, or of how to find relevant information about animal welfare. To be sure, a lack in any of these three types of knowledge may constitute speciesist ignorance; nonetheless, in this article, I will focus on *propositional ignorance*. For one, I assume that most people are ignorant of the fact *that* they have moral obligations towards animals, not that they are ignorant of *how* to act in accordance with those demands. Moreover, I wish to engage with authors in the epistemology of ignorance literature, most of whom discuss propositional ignorance.

Let us take a closer look at propositional ignorance. Propositional ignorance is often defined as a *lack of knowledge* about a proposition (i.e., The Standard View) (e.g., see Le Morvan 2011, 2012, 2013) or as a *lack of true belief* about a proposition (i.e., The New View) (Peels 2010, 2012, 2014, 2016; Peels and Blaauw 2016, Chapter 1). Alternatively, ignorance is defined as a lack of a valuable cognitive state (knowledge or true belief) that is due to *an improper inquiry* (i.e., The Normative Account) (Meylan 2022; Pritchard 2021)*—*where improper inquiry is understood as a missed opportunity to have known or held a true belief, because of one’s own failure to inquire properly (Meylan 2022).

These views can be discussed together as propositional conceptions of ignorance (El Kassar 2018), which hold that S is ignorant of the fact that p, either if S does not know that p, or if S does not have a true belief that p.^6^ For instance, Cheryl Abbate argues that the average meat-eater lacks the belief in the proposition that ‘eating meat is seriously wrong' (2021, p. 68), and she suggests that this could be the reason why people do not adopt vegan diets (Abbate 2021).

Although propositional ignorance has not been discussed with a particular focus on *speciesism*, there are of course particular propositions about which the speciesist could be ignorant. Arguably, the ignorant speciesist does not know about a proposition stating the moral wrongness of speciesism. Let us call this p_sp_. p_sp_ is itself normative in nature,^7^ and denotes that ‘one has the pro tanto moral obligation to act anti-speciesist’.^8^ In turn, the ignorant speciesist does not know that p_sp._

Propositional ignorance allows for further distinctions which can be used to criticize the speciesist. There can be different *kinds* of ignorance, depending on whether one has considered p_sp_ before, and what doxastic attitude one holds about p_sp._^9^If one did consider p_sp_, one could be *disbelievingly* or *suspendingly* ignorant about p_sp_. If one did not consider p_sp_, one could be *unconsideredly* ignorant, *deeply* ignorant, or *completely* ignorant about p_sp_ (Peels and Pritchard 2021). Propositional ignorance thus gives a detailed account of individual ignorance about speciesism.

The Standard View and the New View of ignorance formulate *stative* conceptions concerning the nature of ignorance, which regards ignorance as a cognitive state of not-knowing (Meylan 2022). Alternatively, the Normative Account argues in favour of an *agential* conception concerning the nature of ignorance—i.e., it regards ignorance as a substantive epistemic practice that is ‘essentially actively induced, in the sense that it is part of what it is to be an instance of ignorance to be actively brought about’ (Meylan 2022, sec. ‘Stative vs. Agential Conception of Ignorance’). As such, the Normative Account argues that ignorance is only an appropriate ascription when a negative evaluation of someone’s epistemic standing is warranted—the subject would have known had she properly inquired. In other words, we may only claim that someone is ignorant about a proposition if it entails that *they ought to have* knowledge or true belief about said proposition (Pritchard 2021). For instance, imagine that I do not know a certain pointless truth such as the number of spoons in my cupboard, or an unknowable truth such as the position and momentum of a particle. Rather than claiming I am ignorant of such propositions, it would be more appropriate to say that I simply do not know. Accordingly, an ascription of ignorance has a normative dimension since it entails an intellectual failing of the subject as an inquirer (Meylan 2022; Pritchard 2021).

Several reasons may underlie these intellectual failings. For instance, it has been suggested that ignorance is caused by the individual’s *epistemic vices*, which are ‘character traits, attitudes or thinking styles that systematically obstruct the gaining, keeping or sharing of knowledge' (Cassam 2018a, sec. abstract). Examples of epistemic vices include arrogance, laziness, closed-mindedness, and epistemic insouciance (Cassam 2018b; El Kassar 2018; Medina 2013). If we relate this to speciesist ignorance, it would seem plausible to argue that people either become or remain ignorant of p_sp_ as a result of their epistemic vices. For instance, one could be epistemically arrogant and dogmatic about their belief that animals matter less than humans and that humans are justified to eat meat for a plethora of reasons. Such stringent commitment to speciesist beliefs would surely obstruct knowledge about p_sp_.

Alternatively, it has been argued that intellectual failings manifest ignorance because the agent plays an active role in maintaining her beliefs and *chooses* not to know certain things (Moody-Adams 1994), in order to avoid inconvenient truths (Martín, 2021). Different terms are used for this type of ignorance, such as ‘affected ignorance’, ‘wilful ignorance’, and ‘strategic ignorance’ (for a summary of the different terms used see Onwezen and van der Weele 2016, p. 97). Animal ethicists who discuss this conception of ignorance argue that meat-eaters are motivated to remain ignorant, in order to continue their behaviour (Onwezen and van der Weele 2016; Schwartz 2020; Williams 2008). Although these scholars have not commented on speciesism specifically, they would surely agree that the agent can be wilfully ignorant of speciesism and of her own speciesist actions. Seemingly, Peter Singer alludes to this very idea when he says that ‘ignorance […] is the speciesist’s first line of defense’ (Singer 2015, p. 314).

For the purposes of this article, I regard individual speciesist ignorance to be agential in nature, in line with Duncan Pritchard’s (2021) and Anne Meylan’s (2022) development of the Normative Account. Namely, as mentioned above, p_sp_ is normative in nature because it denotes how one ought to act towards animals. Arguably it is also true that the subject *ought to know* about p_sp_, in order to be able to act in accordance with this moral obligation. Charging someone with speciesist ignorance, then, entails a negative evaluative statement about their epistemic standing, which goes further than saying that someone simply does not know or truly believe p_sp_. This negative evaluative statement also acts on the presumption that had one inquired properly, one would have known about p_sp_. As such, not knowing was a missed opportunity, because one failed to inquire properly (Meylan 2022).

Although I acknowledge that the wilful element plays an important part in many cases of speciesist ignorance, it is not a necessary feature of speciesist ignorance. Granted, we must reasonably assume that at least some instances of speciesist ignorance stem from *unintentional* epistemic failings (e.g., from epistemic laziness).^10^ For instance, someone could simply have failed to consider the ethical issues of eating meat and never have been prompted to do so either. The failure to inquire into p_sp_, thus, need not have stemmed from the *intention* to avoid information and its possible normative implications.

This brings me to the following definition of individual speciesist ignorance:

*Individual Speciesist Ignorance* = _*df*_ S lacks knowledge or true belief about p_sp_, which is due to improper inquiry, where p_sp_ denotes the moral obligation not to discriminate on the basis of species membership in practical deliberations. This ignorance may vary in kind; disbelieving, suspending, unconsidered, deep, or complete.^11^ Moreover, the improper inquiry into p_sp_ may be caused by one’s epistemic vices, and may be wilful.

The propositional and substantive conceptions of ignorance can enrich the animal ethics debate in several ways. First, the different kinds of ignorance that can be ascribed to the speciesist can inform questions on culpability. For instance, perhaps the *deeply* ignorant person is less culpable for her speciesist actions than the *suspendingly* ignorant person. Similarly, motivated ignorance is likely more culpable than non-motivated ignorance. Second, the different kinds of individual ignorance are important to consider for effective animal advocacy. If someone is *unconsideredly* ignorant, for instance, it may be sufficient to simply make her aware of the target proposition p_sp_. Alternatively, someone who is *disbelievingly* ignorant may require more convincing to believe p_sp_. Third, affected ignorance conceptions explain why so many people are ignorant as a result of the benefits they receive from not knowing. And it explains the form and contents that ignorance takes (Martín 2021).

Although this conception of individual speciesist ignorance already provides many of the aforementioned benefits that an epistemology of ignorance promises for the animal ethics debate, this definition by itself does not have satisfactory descriptive accuracy. To start, there may be cases of ignorance which cannot be clearly attributed to faulty cognitive practices, nor wilful ignorance (Martín 2021); in such cases, ignorance ascriptions might be inappropriate.^12^ Moreover, the individual conception fails to explain why speciesist ignorance (and the consequential wrongdoings) is so widespread, i.e., ‘the ways in which so many individuals […] have tended to converge on the same forms of ignorance over time’ (Martín 2021, p. 871). Namely, it is not satisfactorily explained *how* one’s ignorance about p_sp_ comes about to begin with. There must be reasons why it is not purely accidental that *the majority of people* are ignorant about p_sp_. And, perhaps most importantly, it is questionable whether these are appropriate accounts with which to discuss speciesism as a form of discrimination. Speciesism is, arguably, the result of many social processes, and so requires analysis of how it manifests in social institutions and relationships (Nibert 2002). Indeed, our experiences, desires, and beliefs are influenced by our social, political, historical, and physical environment (Woomer 2019). Moreover, the agent’s situatedness (i.e., her knowledge, skills, environment, and so forth) defines what she is able to know and what she is ignorant about in a given context (Alcoff 2007). Therefore, any discussion of how ignorance and speciesism relate, must additionally look *further than an individualist epistemology* that focuses on the individual cognizer (Mills 2007)*.*

Alternatively, a *social epistemology* of ignorance—unlike traditional epistemology—focuses on ‘social paths or routes to knowledge, […] does not restrict itself to believers taken singly, [… and] addresses the distribution of knowledge or error within the larger social cluster’ (Goldman 1999, p. 4). By taking the social dimensions of ignorance seriously, it becomes clear how ignorance and discrimination intersect and why individual ignorance comes about to begin with (Mills 2007). As a first move in this direction, I discuss whether speciesist ignorance can, additionally, be understood as a form of *group ignorance*. Subsequently, I consider how speciesist ignorance is encouraged through social structural processes.

## Group Ignorance

In order to claim that a group is ignorant *as a group*, two conditions must be met. First, a significant part of its operative members must be ignorant about p, and second, this individual ignorance results from a group dynamic—‘such as group agency, collective epistemic virtues or vices, external manipulation, lack of time, interest, resources, or information, or a combination of these' (Peels and Lagewaard 2022, p. 14). It does not matter if one member knows, as long as the group remains ignorant *as a group* (Peels and Lagewaard 2022). Group ignorance is not just the aggregate of individual ignorance, since the group *itself* can be assigned a certain group dynamic, such as epistemic vice: e.g., ‘it is not the result of epistemic bad luck but the result of epistemic vice' (Peels and Lagewaard 2022, p. 7). Here, I understand collective epistemic vice—in analogy with individual epistemic vice—as a group’s character traits, attitudes, or thinking styles which systematically obstruct the creation, sharing, and storing of knowledge. I assume that groups may display collective epistemic vice and virtue *as a group,* which cannot be reduced to the epistemic vices of its individual members (e.g., Baird and Calvard 2019; De Rooij and de Bruin 2022; Fricker 2020; Lahroodi 2007; Meyer 2023). Such group ignorance is not a lack of something, but the presence of substantive epistemic practices that serve the interest of the dominant group (Alcoff 2007). Correspondingly, we can discuss the group’s mistaken beliefs (Mills 1997, 2007) and epistemic vices (Medina 2013; Peels and Lagewaard 2022), group-level moral obligations, and group-level culpability (de Haan 2021).

While assuming that Peels and Lagewaard’s account of group ignorance (and accounts of collective epistemic vice) is broadly correct, I will argue that speciesist ignorance indeed qualifies as group ignorance. First, just as I assumed above that the average speciesist *individual* is morally ignorant, I assume here that the *group* of speciesists (whether in a particular society or globally) comprises a majority of members who are morally ignorant about p_sp_. Admittedly, not everyone is ignorant about p_sp_. However, homogeneity need not be a property of group ignorance: the group can be constituted of members who experience different kinds of ignorance (ranging from disbelieving to complete ignorance), and the group may also have members who are not ignorant.^13^ Second, individual speciesist ignorance indeed results from a group dynamic. To substantiate this, I propose that the group has *a collective interest* in maintaining speciesism through ignorance, and that the group exhibits *collective epistemic vice*.

Continued speciesism stands to benefit humans enormously. Consider how our interests frequently outweigh animals’ interests, in order to allow us to use animal products for our food, clothing, and leisure (e.g., companionship, entertainment, tourism, hunting, and so forth). Surely, an anti-speciesist commitment would require vast changes in our lives and society as we know it, and push our abilities to be flexible in changing various cultural traditions and norms. It seems a relatively uncontroversial claim, then, that humans have a collective interest in continuing the speciesist status quo, and to do so by maintaining speciesist ignorance.

In this vein, social epistemologists stress the importance of considering *who* can afford to stay ignorant about injustice and who cannot. Particularly, staying unaware of certain injustices is enjoyed from a position of *privilege,* by the socially dominant group. Relatedly, it has been argued that the socially dominant group harbours epistemically viceful practices, because one’s membership of a certain dominant social group can leave one ‘epistemically disadvantaged’ (Alcoff 2007). For instance, it is argued that the socially dominant group has a positive interest in misinterpreting the world, which gives rise to its epistemic vices such as ‘epistemic arrogance’ and ‘epistemic laziness’: because one  neither has a *need to know* nor an *interest to know* about what privilege involves, in many cases this leads to epistemic blind spots which deteriorate one’s epistemic character over time and manifest in epistemic vice (Medina 2012). This means that ‘there are identities and social locations of sorts, that are in some cases epistemically disadvantaged or defective' (Alcoff 2007, p. 40). Arguably, the most well-known scholar for using this argument is Charles Mills, who argued that race and white privilege play a significant causal role in maintaining racial injustices (Mills 1997, 2007).^14^ Mills himself admits that there are other types of privileged, group-based ignorance, such as male ignorance (Mills 2007).^15^ Who, then, should be included in the group that is under the spell of *speciesist* ignorance?

I will argue that humans (in speciesist societies) experience ‘human privilege’ opposite non-human animals, and that this privilege gives rise to collective epistemic vices which implicate our knowledge about animals specifically. In doing so, I additionally define a group-based speciesist ignorance. Namely, taken together, a collective interest in continued speciesism and collective epistemic vices would differentiate the group which exhibits speciesist ignorance.

There is agreement in the literature that privilege contains the following five components:^16^

First, privilege is a special advantage; it is neither common nor universal. Second, it is granted, not earned or brought into being by one’s individual effort or talent. Third, privilege is a right or entitlement that is related to a preferred status or rank. Fourth, privilege is exercised for the benefit of the recipient and to the exclusion or detriment of others. Finally, a privileged status is often outside the awareness of the person possessing it. (Black & Stone 2005, p. 244)

Ironically, the privileged are often unaware of their own privilege while simultaneously believing that their advantage is deserved and that the disadvantage of the non-privileged is in some way the latter’s own fault (Black and Stone 2005). If this attitude remains unchecked and unchallenged, it results in the oppression of the non-privileged (Black and Stone 2005; Choules 2007). As mentioned above, some social group identities make it so that one is epistemically disadvantaged and wrongly interprets information. Membership of a certain dominant social group encourages these epistemic failures along with the consequent ignorance.

Pursuant to these five components, I wish to identify *species membership* as another domain of privilege.^17^ Indeed, humans experience privilege in relation to animals. Speciesism guides much of our use of animals. As a result, we use various sentient animals in ways that we would never even consider using humans. We do not go to human zoos (anymore), we do not eat human meat, nor do we wear human leather (Jaquet 2022). Yet humans enjoy the privilege of unquestioningly using animals for exactly those purposes. Human interests override the interests of animals—an advantage which is not earned but granted simply by one’s group-membership of the human species. This privilege is exercised for the benefit of humans, which structurally disadvantages animals. In reference to the final feature of privilege, my exploration of the relation between ignorance and speciesism shows that all previous features often fall outside of the awareness of the person possessing the privilege.^18^

Human privilege—like any type of privilege—gives rise to collective epistemic vices, such as epistemic laziness and arrogance. An example of human privilege would be that the practice of eating meat has always been a part of our diet. The practice of eating meat is considered normal; i.e., we commonly eat meat for breakfast, lunch, and dinner, and at festivities. Since the majority of people enjoy these privileges, as a member of the socially dominant group, we have no need to know and very little interest or incentive to reconsider our moral commitments concerning eating meat and the human-animal-relationship. The epistemic costs which we would have to invest to inquire into p_sp_ are high, especially if we also have to deal with uncomfortable information and with the moral implications of knowing about p_sp_. Instead, concerning animal-related issues, it is much less epistemically costly to preserve our privilege, by engaging in defective epistemic reasoning—e.g., such as identity-protective reasoning,^19^ dogmatism, narrow-mindedness, and intellectual laziness (Peels and Lagewaard 2022). Such collective epistemic vices play a significant role in the collective avoidance and downplaying of information about our unfair social advantage opposite animals. In sum, the collective interest in maintaining speciesism and the collective faulty epistemic behaviour concerning animal-related issues characterizes the ignorant group. Thus, speciesist ignorance can be regarded as a group ignorance.

My restriction to ‘human privilege’ allows for the possibility that not *all* humans on the planet are part of a group that by default exhibits epistemic faulty behaviour concerning speciesist beliefs. Perhaps there exist peoples which do not exhibit faulty epistemic behaviour in order to harbour collective speciesist belief. Admittedly, in such societies speciesist ignorance would not exist as a group-based ignorance, although surely individual instances of speciesist ignorance could still be present. However, as long as the aforementioned components of (human) privilege are largely applicable in a certain society, speciesist ignorance is not only individual but also distinctly group-based in nature. The label *privilege* highlights that the group has a positive interest in keeping this status, and the label *human* privilege highlights why the related epistemic vices would lead to *speciesist* ignorance in particular.

This brings me to the following definition of group-based speciesist ignorance:

*Group-Based Speciesist Ignorance* = _*df*_ Due to a group’s epistemic vices that result from human privilege, a significant number of a group’s operative members lack knowledge or true belief about their moral obligation not to discriminate on the basis of species-membership in practical deliberations. The operative members’ ignorance can vary in kind (i.e., disbelieving, suspending, unconsidered, deep, and complete),^20^ and any or all these kinds of ignorance may be present.

For the purposes of animal ethics, the group-based conception of speciesist ignorance offers several benefits over the individual conception. First, it does not merely state *that* individual speciesist ignorance is widespread; it also explains *why* this is the case. Second, not all speciesism can be explained by individual ignorance—in other words, not all speciesist wrongdoings result from individual ignorance about p_sp_. To reiterate, although such cases may undermine ascriptions of individual ignorance, they do not belie the existence of group-based speciesist ignorance. Consider someone who *defends* the claim that speciesism is morally defensible. It is likely unproductive to claim that this person is ignorant of p_sp_. To do so might stretch the individual account of ignorance too thin, in order to encompass all speciesist beliefs. Whereas the *individual* conception of speciesist ignorance fails to capture these instances of continued speciesism, the *group-based* conception of ignorance does not, since the latter exists even when some members are not ignorant of p_sp_. Any allegedly non-ignorant speciesist still participates in the social processes that undermine the moral importance of animals and harbour collective speciesist beliefs. Although I showed that ignorance can be captured by the individual conception perfectly well, this conception alone cannot sufficiently explain why this ignorance about a form of discrimination is so socially widespread.

Lastly, the group-based conception of speciesist ignorance is useful to the animal ethics debate as an analytical tool for critiquing continued speciesism as a *collective* wrongdoing towards animals.^21^ Focusing on collective wrongdoing, rather than individual wrongdoing, is in line with the field’s ‘political turn’, which champions justice for animals (for a summary of this political turn, see Milligan 2015). Animal ethicists are discussing, for instance, whether animals deserve labour rights (Blattner et al. 2019; Cochrane 2016), citizenship (Donaldson and Kymlicka 2011; Hooley 2018; Kymlicka 2017), or a form of political representation (Driessen 2014; Hooley 2018; Meijer 2016, 2017). An account of ignorance that is not purely individual better reflects the political turn towards *collective* wrongdoings and best explains its intricate relation with speciesism as a structural injustice.

To be clear, my conception of group-based speciesist ignorance is not a full rejection of individualist epistemology. Individual conceptions of speciesist ignorance should not be disregarded, since the different *kinds* of ignorance still impact questions of culpability and effective animal advocacy strategy to actively reject ignorance.^22^*Taken together*, individualist and social epistemology render a comprehensive epistemology of speciesist ignorance.

One could object that my conception of speciesist ignorance is not a unified account, as it allows for both individual and group-based conceptions of ignorance. I would respond that there can be two levels to this phenomenon, and so there is no need to reduce one to the other. A two-dimensional view of speciesist ignorance is even advantageous, as it provides both descriptive accuracy about speciesists’ ignorance, as well as explanatory power about why this ignorance is widespread. By contrast, a purely group-based account would not be able to account for the former, and a purely individual account would not be able to account for the latter.

## Ignorance Production and Perpetuation of Injustice

With this understanding of *what speciesist ignorance is*, we should further discuss what epistemic circumstances *produce it*,^23^ in order to fully comprehend its normative implications within animal ethics, and to be able to remedy its negative consequences. Together with individual and collective faulty epistemic inquiry, I will argue that s*ocial structural processes* incentivize individual and group-based speciesist ignorance. Admittedly, individuals and groups both play a role in maintaining their own ignorance—i.e., through intentional and/or unintentional faulty epistemic behaviour. However, we must additionally consider the epistemic climate which allows for this maintenance of ignorance to occur successfully, and even incentivizes its maintenance. Understood broadly, ‘social structural processes’ include ‘both institutions […] and culture, or networks of cultural schemas (e.g. beliefs, concepts, attitudes) […], as well as the social norms and practices that emerge from widespread internalization of these schemas' (Martín 2021, p. 876). Martín argues that social structural processes systematically give rise to injustice, and that they do so in part by encouraging ignorance (Martín 2021). Similarly, I present various social structural processes which systematically give rise to injustices towards animals, and maintain that they do so—at least in part—by encouraging individual and group-based speciesist ignorance. This promotion of ignorance, falls into two categories: i) impeding agents’ *accessing* information which they need to know about p_sp_, and ii) impeding agents’ appropriately *interpreting* information in order to know that p_sp_.^24^

First, agents are structurally impeded in *accessing* information. Most obviously, animals are literally made invisible insofar as they are hidden from people’s view. Farm protection bills make it almost impossible to reveal information about what happens on farms (Broad 2016; Lin 2015; O’Sullivan 2014). Moreover, animals are invisible in the products we eat, which are, for instance, presented in appealing packages. Similarly, when eating meat, its animal origin is disguised by cutting it into smaller pieces (Nussbaum 2003). Animal products such as fish sticks, chicken nuggets, or hotdogs do not resemble the sentient animals from which they derive—and this appearance keeps people ignorant about the link between food consumption and animal welfare (Cairns and Johnston 2018). Students and researchers have to sign confidentiality contracts which prevent them from speaking out against the mistreatment of animals or communicating this information to other parties. To that end, ‘procedures for animal experimentation are protected by intellectual property rights and the right to freedom of research’ (Blattner 2019, p. 293). It has been suggested by several animal ethicists that this invisibility is partly the cause for non-responsiveness to injustices towards animals (for instance, by Acampora 2016; O’Sullivan 2011; Williams 2008). Further, the epistemic resources of animal advocates are often discredited, which unfavourably influences people’s willingness to listen to their arguments and thus to *access* relevant information about speciesism. Negative attitudes towards vegans and vegetarians are common (Earle et al. 2019; Horta 2018), such as the view that vegans are ‘killjoys’ (Twine 2014). Newspapers perpetuate derogatory stereotypes of veganism, describing it as impossible to sustain or as a fad diet, while characterizing vegans as oversensitive or hostile individuals (Cole and Morgan 2011). It has been argued that such dismissal of animal rights advocates as ‘crazy’ or irrational diminishes their social justice claims (Wrenn et al. 2015) and functions as a cultural reproduction of speciesism (Cole and Morgan 2011).

Second, agents are encouraged to *interpret* speciesist uses of animals as morally permissible. Animals are also made figuratively ‘invisible’ in the language that we use to talk about them. This language allows us to dissociate from the sentient animal in a way which carries normative weight. For instance, by eating ‘beef’ we are not aware of the cows (Adams 2018); in turn, this framing impacts their welfare, since animals are framed as products instead of sentient beings who experience complex lives (Buller and Roe 2012). This happens in animal agriculture, but also in animal research, where the animal is reduced to a product of laboratory work (Lynch 1988). Because our language does not reflect the lived experience of these sentient beings, this affects the agent’s ability to access evidence about animals and to recognize their suffering (Williams 2008). Even if they are not literally invisible to us (i.e., we go to the zoo, eat meat, and drink milk), we cannot figuratively see them as having rich subjective lives. It has also been argued that animals are not of direct concern and thus ‘invisible’ under animal law, which—ironically—instead concerns the *human* owners, caretakers, or perpetrators of acts against animals. And thus, ‘when these forms of jurisdiction are exercised, animals themselves remain invisible, which reinforces the view that they do not matter' (Blattner 2019, p. 233).

Moreover, public policy and legislation implicitly reflect the dominant view that animals can legitimately be used for human purposes. Such policies justify using animals for various reasons, such as feeding people, curing disease, entertainment, and education (Stallwood 2017). And this encouragement is explicit. Consider advertising for ‘humane' alternatives to regular animal agricultural organizations, such as free-range or organic farms, where the customer is promised farming with a conscience (Acampora 2016). Campaigns such as this ensure that people can buy factory-farmed products, while simultaneously feeling responsible for the environment (Schwartz 2020). Not only meat lobbyists have this effect. Also welfare-labelling motivates customers to make the ‘most' animal-friendly choice, which completely obscures the fact that the most animal-friendly option would be to leave out meat altogether. Further, it has been argued that think tanks produce ignorance about the link between dietary choices involving animal products and climate change (Almiron et al. 2021).

These impediments to *accessing* and appropriately *interpreting* information about animals (and about p_sp_) are clearly successful. Indeed, a recent study showed that one is not born a speciesist, but rather one cultivates speciesist attitudes over one’s lifetime. The authors compared the attitudes towards different sorts of animals from children from ages nine through ten, young adults from ages 18 through 21, and adults from ages 29 through 59 (McGuire et al. 2022). They found that children showed less speciesism, that they were less likely to categorize a farm animal as food, that they did not believe that pigs ought to be treated differently than humans or dogs, and that they deemed eating meat less morally permissible (McGuire et al. 2022). The fact that speciesist attitudes are cultivated over a lifetime suggests that the individual speciesist is encouraged to fail epistemically and to remain ignorant.

The abovementioned examples of social structural processes do two things. First, they substantiate my definition of group-based speciesist ignorance, by showing that speciesist ignorance is often the effect of instilled practices common to the group. Second, they show how various social structural processes *produce* speciesism and speciesist ignorance. Namely, by discouraging people to access and interpret information related to animals, structural social processes systematically obstruct the gaining, keeping, or sharing of knowledge concerning anti-speciesism. Thus, although speciesist ignorance is *defined* by improper (individual or collective) inquiry, we should not lose sight of the structures (i.e., social norms and practices, as well as, mechanisms, systems, and institutions) that stimulate faulty epistemic behaviour and enable us to maintain speciesist ignorance.^25^

Furthermore, in what follows, I argue that speciesist ignorance, in turn, *perpetuates* injustice towards animals—i.e., speciesist ignorance is a *facet* of injustices towards animals. To illustrate how ignorance serves to perpetuate injustice exactly, consider Alcoff’s structural argument (2007). The structural argument consists of three steps:

1. Collective wrongdoings in society are not acknowledged to be morally wrong; thus, there is a dominant view that the society is basically fair and just.
2. There is likely evidence against this dominant view, which is potentially visible to everyone.
3. Yet the assessment of this countervailing evidence is regularly dismissed to uphold the dominant view (all three steps paraphrased from Alcoff 2007, p. 48).

The socially dominant group, according to Alcoff (2007), has a positive interest in misinterpreting the world, which feeds faulty epistemic practices (such as dismissing evidence). Her structural argument shows that the presence of a particular type of ignorance allows its related injustice to perpetuate, by reinforcing the ignorance that occurs in society about certain wrongdoings. Consequently, we may claim that ignorance is a *facet* of the injustice it perpetuates. The same holds true, I claim, for speciesist ignorance.

Speciesist ignorance follows all three steps of the structural argument. First, the speciesist society is unjust towards animals, without a widespread acknowledgement of this. Second, as the field of animal ethics and the animal advocacy movement have shown, there is sufficient evidence of wrongdoings towards animals that is potentially visible to the general public and the individual epistemic agent. Third, proper cognitive assessment of the evidence of animal injustice is dismissed by restricting *access* to information and by discouraging the *interpretation* that the dominant view is actually morally impermissible. The dominant view, in this case, proclaims that the human-animal-relationship as it stands is just. Others have identified this ‘carnist’ ideology, which ensures that speciesist animal use is seen as ‘normal’ and that injustices towards animals continue unquestioningly (Acampora 2016; Joy 2020). By focusing on *ignorance*, I have provided a novel explanation of how injustices towards animals and the accompanying carnist ideology are sustained. Namely, speciesist ignorance serves to *perpetuate* its corresponding injustice.^26^ It does not suffice to say that ignorance is an obstacle to injustice; rather, it plays an active role in its continuation and is thus a *facet* of injustice (see Alcoff’s first point). This is not to say that *all* instances of animal injustices are explained by speciesist ignorance, but rather that all animal injustices are perpetuated by speciesist ignorance, even if they are not *caused* by speciesist ignorance.

The structural argument increases the value of the notion of speciesist ignorance for animal ethics even further. Namely, insofar as ignorance is a facet of injustice, any normative project which aims to transition towards justice must reflect on the *epistemic circumstances* within society which harbour ignorance practices that obstruct this transition. Relatedly, we should question which epistemic circumstances *are conducive with* knowledge about p_sp_ and thereby facilitate a transition towards inter-species justice. Any attempt which focuses solely on improving the epistemic behaviour of individuals and groups will be insufficient to solve the widespread issue of speciesist ignorance. Without tackling the various causes of ignorance, any attempt at fighting injustice will most likely be in vain. On the plus side, insofar as speciesist ignorance is a facet of injustices towards animals, remedying ignorance will automatically contribute to the very dismantling of this injustice, and is not a mere preparatory step for a transition towards inter-species justice.

## Conclusion and Outlook

My purpose in this article was to introduce the epistemological concept of *speciesist ignorance* into the animal ethics debate. I explained why the particular epistemic relationship between humans and non-human animals is rife with epistemic misuses, whereby we interestedly wield our uncertainties and biases against animals. This called for a philosophical analysis of the connections between ignorance and speciesism. Accordingly, I provided definitions of both individual and group-based speciesist ignorance. Although speciesist ignorance does not cause *all* speciesist wrongdoings, it is a *facet* of animal injustice, because it perpetuates itself and sustains injustice.

In sum, my epistemological account of speciesist ignorance can enrich the animal ethics debate in several ways. To begin with, it provides epistemic value insofar as it more aptly describes how ignorance about speciesism manifests itself and how it perpetuates injustices towards animals. Moreover, the account influences the conditions under which speciesist moral wrongdoings are blameworthy, which should in turn spark debate about the epistemic duties that individuals and institutions have to prevent and remedy speciesist ignorance. Furthermore, insofar as this conception explains how injustice towards animals is perpetuated, it can suggest which animal advocacy strategies may be most effective to remedy speciesist ignorance and related animal injustices.

## Footnotes

1 I deliberately employ the term ‘*anti*-speciesism’, rather than ‘*non*-speciesism’, because the anti-speciesist duty entails an *active* rejection of institutional and structural speciesism. I base this choice of terminology on an analogy with critical race theory, where non-racism and anti-racism are distinguished: the former is a passive rejection of extreme racism; the latter is an active rejection of institutional and structural racism. The merely passive rejection does little to transform racist thinking and may even reinforce racial structures. See (King 2016; King and Chandler 2016).

2 Admittedly, animal ethicists have employed different definitions of speciesism. This choice, in turn, can influence whether speciesism is unjustified by definition or whether it can function as a neutral descriptive term. It falls beyond the scope of my article to weigh which definition is better and to justify why this is so. For argument’s sake, I will simply use Oscar Horta’s definition in (Horta 2010), which Francois Jaquet has convincingly supported in (Jaquet 2022).

3 A few scholars do discuss ignorance as a separate obstacle within animal ethics, but they do not comment explicitly on its relationship to speciesism. For instance, (Abbate 2020, 2021; Acampora 2016; Fischer 2020; Kahn 2011; Onwezen and van der Weele 2016; Schwartz 2020; Williams 2008).

4 Of course, an epistemology of ignorance only covers a small portion of the epistemic dimensions of speciesism, including: working through moral disagreement about the wrongness of speciesism and assessing what this requires for the reconfiguration of the human-animal-relationship; explaining what it means to *know* about one’s moral obligations towards animals; determining under what circumstances we possess sufficient *know*-*how* to act in anti-speciesist ways; specifying epistemic harms or wrongdoings related to animal injustices—i.e., towards animals and/or animal advocates, etc.

5 Other distinctions include: stative vs. agential; individual vs. group ignorance; structural vs. non-structural ignorance. See below.

6 For an appropriate ascription of ignorance, two conditions must be met, namely that (i) p is true, and (ii) S is an epistemic agent (Nottelmann 2016). For S to be an epistemic agent, she must have sufficient epistemic tools with which she can inquire (e.g., see Woomer 2019), and she must also find herself in an environment with the conceptual resources to inquire into p (Peels and Pritchard 2021).

7 As such, propositional ignorance about speciesism concerns a type of moral-ignorance, i.e., ‘ignorance about the moral impermissibility of one’s conduct' (Wieland 2020, p. 2). To illustrate, a non-moral proposition could be the temperature of my cup of coffee, or the time at which my train will arrive.

8 I regard this as a *pro tanto* obligation, which can be overridden under special circumstances—namely, all things considered, we might not have this moral obligation depending on contextual considerations.

9 This stems from the widely held premise in analytical epistemology that there exist exactly three doxastic attitudes; i.e., belief about p, disbelief about p, and suspension of judgement about p (Peels 2019).

10 At this time I do not provide additional arguments to defend the Normative Account. I presuppose this account is broadly accurate and leave a complete defence for a later time. The debate on the nature of ignorance should not distract from the aim of the paper, which is to introduce an epistemological account of speciesist ignorance and thereby more adequately criticize sustained structural speciesism and injustices towards animals. The debate between stative and agential accounts should not be overstated, since they can both discuss the crucial normative implications of agential features even though they disagree on the nature of ignorance (Peels 2019).

11 Objectual ignorance and practical ignorance should also be further considered for a complete rendering of individual speciesist ignorance. As I explained before, this is beyond the article’s scope.

12 Moreover, at some point deep ignorance and suspension of judgement become irrational. Irrational ignorance falls outside of the scope of this article. Still, it is important to recognize that at some point, it is inappropriate to call the speciesist agent ignorant.

13 This implies that, just as individual ignorance comes in degrees (Brogaard 2016; Nottelmann 2016), so may group ignorance (Peels and Lagewaard 2022). This possibility leaves room to argue that one society has speciesist ignorance to a higher degree than another society.

14 Mills defends a *privileged, group-based white ignorance*, in which *race* plays a causal role in racial injustice towards non-white people (Mills 1997, 2007). Many have discussed white ignorance—i.e., the way that ignorance and racial discrimination relate (Alcoff 2007; Bailey 2007, 2017; Bain 2018; Fricker 2013; Fricker et al. 2016; Martín 2021; Medina 2012, 2013; Outlaw 2007; Spelman 2007; Sullivan 2007; Sullivan and Tuana 2007; Woomer 2019).

15 The analogy between white ignorance and speciesist ignorance lies in the *pattern* of behaviour that gets encouraged by social and political structures along with the way in which this pattern produces ignorance and perpetuates injustice. Importantly, however, it does *not* equate Black oppression with animal oppression.

16 To illustrate, white privilege (in reference to white ignorance) manifests in many ways. A few examples are the following; ‘I can, if I wish, arrange to be in the company of people of my race most of the time. [and] Whether I use checks, credit cards or cash, I can count on my skin colour not to work against the appearance that I am financially reliable. [and] I can criticize our government and talk about how much I fear its policies and behaviour without being seen as a cultural outsider' (McIntosh 2018, p. 12).

17 Human privilege is not a frequently used term in animal ethics. For instance, Ralph Acampora does use it, but does not provide a clear analysis or definition. See (Acampora 2016).

18 One could object that the concept of privilege does not translate so easily. For instance, the badness of privilege relies on the underlying idea of comparability—i.e., it is generally accepted that human beings are comparable and deserving of the same treatment. Therefore, a position of privilege that puts a group at an advantage is somehow wrong. Humans and other animals differ more; nonetheless, I assume that their sentience grounds their equal moral consideration. As such, if one accepts my starting assumption, this should not hold one back from buying in to the argument that species can be a domain of privilege.

19 Peels and Lagewaard draw inspiration from Kahan’s notion of ‘identity protective cognition’ (2017), where he refers to the habit of individuals from a certain social group to be more likely to accept identity-affirming misinformation, rather than identity-threatening information, which affirms the dominant beliefs of the group.

20 It should be noted that objectual ignorance and practical ignorance should also be considered further for a complete rendering of group-based speciesist ignorance.

21 To put this into perspective, most authors who discuss ignorance in reference to animal ethics focus on *individual* culpability; see (Abbate 2020; Fischer 2020; Guerrero 2007; Williams 2008).

22 Questions of culpability and practical solutions fall outside the scope of this article. Still, I mention them because they show that individual ignorance is important to consider, even though speciesist ignorance can be conceptualized as a form of group ignorance. The group members’ different kinds of individual ignorance constitute the group ignorance.

23 The field of Agnotology analyses the production of ignorance, and discusses how ignorance creates or sustains social injustices. For instance, for various agnotological works on different topics, see (Proctor and Schiebinger 2008).

24 This is based on Lauren Woomer’s distinction between epistemic *access* tools and epistemic *interpretative* tools. See (Woomer 2019).

25 Some go as far as to argue that ‘structural ignorance’ only qualifies as an instance of ignorance because of these social structures (e.g., Alcoff 2007; Martín, 2021; Santos 2021). For the purposes of this article, I do not regard ‘structural’ as a feature pertaining to the nature of ignorance. Instead, I discuss structural encouragement as a significant causal feature of speciesist ignorance.

26 This is a crucial element of Mills’s argument about white ignorance. He states that white ignorance serves to ‘reproduce this racial order, securing the privileges and advantages of the full White citizens and while maintaining the subordination of nonwhites’ (Mills 1997, p. 14).
